# Conventional Coronary Angiography Induced Takotsubo Cardiomyopathy Complicated with Cardiac Tamponade

**DOI:** 10.1155/2017/5631264

**Published:** 2017-03-01

**Authors:** Min Gyu Kang, Kye-Hwan Kim, Jin-Sin Koh, Young-Hoon Jeong, Jin-Yong Hwang, Jeong Rang Park

**Affiliations:** ^1^Division of Cardiology, Department of Internal Medicine, Gyeongsang National University and Gyeongsang National University Hospital, Jinju, Republic of Korea; ^2^Division of Cardiology, Department of Internal Medicine, Gyeongsang National University and Gyeongsang National University Changwon Hospital, Changwon, Republic of Korea

## Abstract

Takotsubo cardiomyopathy (TCM) is a transient left ventricular dysfunction that typically occurs after emotional or physical stress. TCM has a benign prognosis and serious complications are uncommon. However, though very rarely reported, cardiac tamponade has occurred on some occasions. We hereby report the case of a 70-year-old woman who underwent coronary angiography with an ergonovine provocation test to evaluate recurrent chest pain and was readmitted 7 days later presenting with TCM, followed by left ventricular outflow tract obstruction and cardiac tamponade.

## 1. Introduction

Takotsubo cardiomyopathy (TCM) is an acute cardiac syndrome characterized by transient and reversible systolic dysfunction of the left ventricle (LV) [1]. TCM is often misdiagnosed as acute coronary syndrome (ACS) [2]. TCM predominantly affects elderly women and is uniquely preceded by emotional or physical stress [3, 4]. Various stressors have been reported to induce TCM, including minimally invasive procedures such as pacemaker placement [5]. The prognosis is benign; however, there are serious complications such as life-threatening arrhythmia, left ventricular outflow tract obstruction, acute heart failure, and cardiac death can follow [1]. Cardiac tamponade has also been reported on very rare occasions [6]. In this report, we present an unusual case of TCM induced by coronary angiography (CAG) with an ergonovine provocation test that was complicated with cardiac tamponade.

## 2. Case Presentation

**Figure 1:**
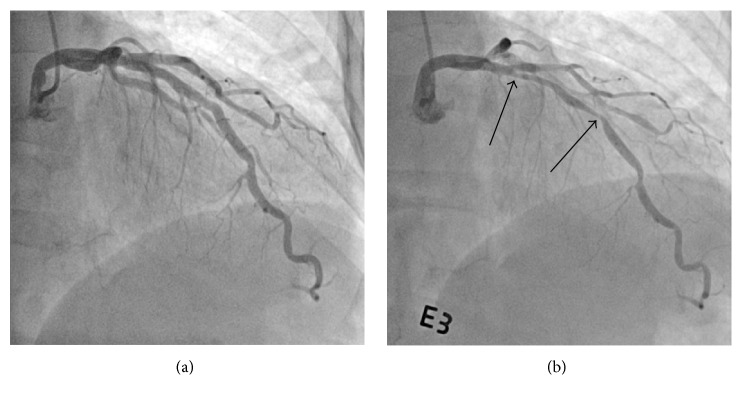
(a) Coronary angiography showed atherosclerotic change with mild stenosis in the left anterior descending artery. (b) Ergonovine provocation induced coronary spasm in the mid-left anterior descending artery (arrows), causing up to 70% stenosis as demonstrated by quantitative coronary analysis.

A 70-year-old woman, presenting with vague chest pain that she had been experiencing for the past year, was admitted for coronary angiography. She did not have any cardiovascular risk factors. There were no abnormal findings during a physical examination or during any routine tests, which included electrocardiogram (ECG), chest X-ray, blood tests, and transthoracic echocardiography (TTE). CAG showed atherosclerotic change with mild stenosis in the left anterior descending artery (LAD) (Figure 1(a)). Provocation test (60 *μ*g of ergonovine in physiological saline into the left coronary artery over a period of 3 minutes) showed coronary spasm in the mid-LAD causing up to 70% stenosis by quantitative coronary analysis (Figure 1(b)). Typical chest pain and T wave inversion were recognized simultaneously. She was diagnosed with vasospastic angina and discharged with vasodilators (a calcium channel blocker and nitrate).

**Figure 2:**
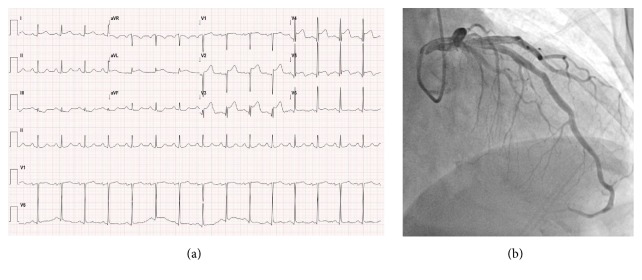
(a) Electrocardiography showed ST segment elevation in precordial leads (V2~V6). (b) Coronary angiography revealed neither spastic occlusion nor significant narrowing of coronary arteries.

**Figure 3:**
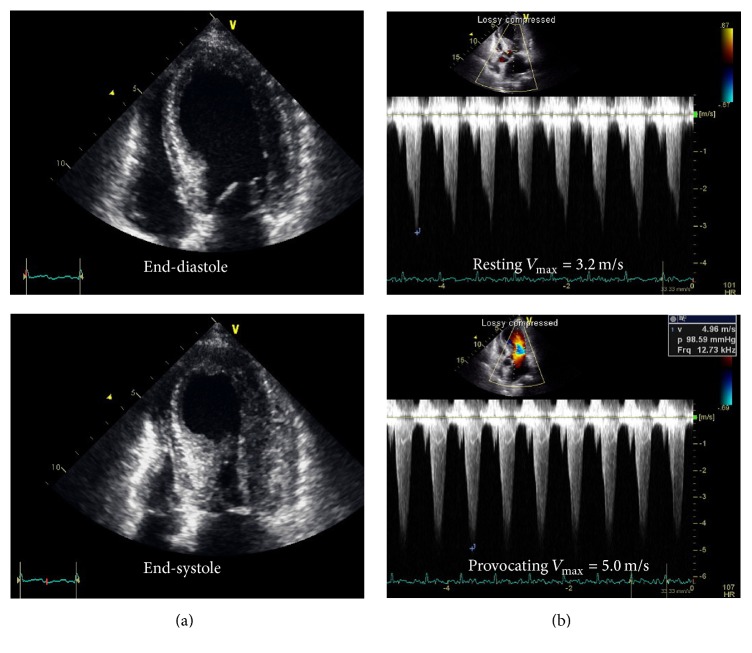
Transthoracic echocardiography showed an apical hypokinesia with normal left ventricular systolic function (a). Left ventricular outflow tract obstruction was confirmed by color flow acceleration with high-pressure gradient; maximal velocity (*V*_max_) 3.2 m/s at resting and 5.0 m/s after Valsalva maneuver (b).

One week after her discharge, she was readmitted to the emergency department presenting with continuous substernal chest pain unfavorable response to the vasodilators. Initial ECG showed ST segment elevation in the precordial leads (V2~V6), and ST segment elevation persisted after intravenous infusion of nitroglycerin (Figure 2). The patient was transferred to a cardiac catheter laboratory, but there was neither occlusion nor significant narrowing on coronary arteries (Figure 2). Intravascular ultrasound confirmed an eccentric soft plaque without plaque rupture or thrombus formation. TTE showed an apical hypokinesia with normal range of LV ejection fraction and LVOT obstruction (Figure 3). Blood tests revealed elevated levels of the serum cardiac enzymes creatine kinase-MB (31.5 ng/mL; normal range < 3.6 ng/mL) and troponin I (9.83 ng/mL; normal range < 0.16 ng/mL). The patient was diagnosed with TCM caused by previous CAG with ergonovine provocation test and was admitted for close observation.

**Figure 4:**
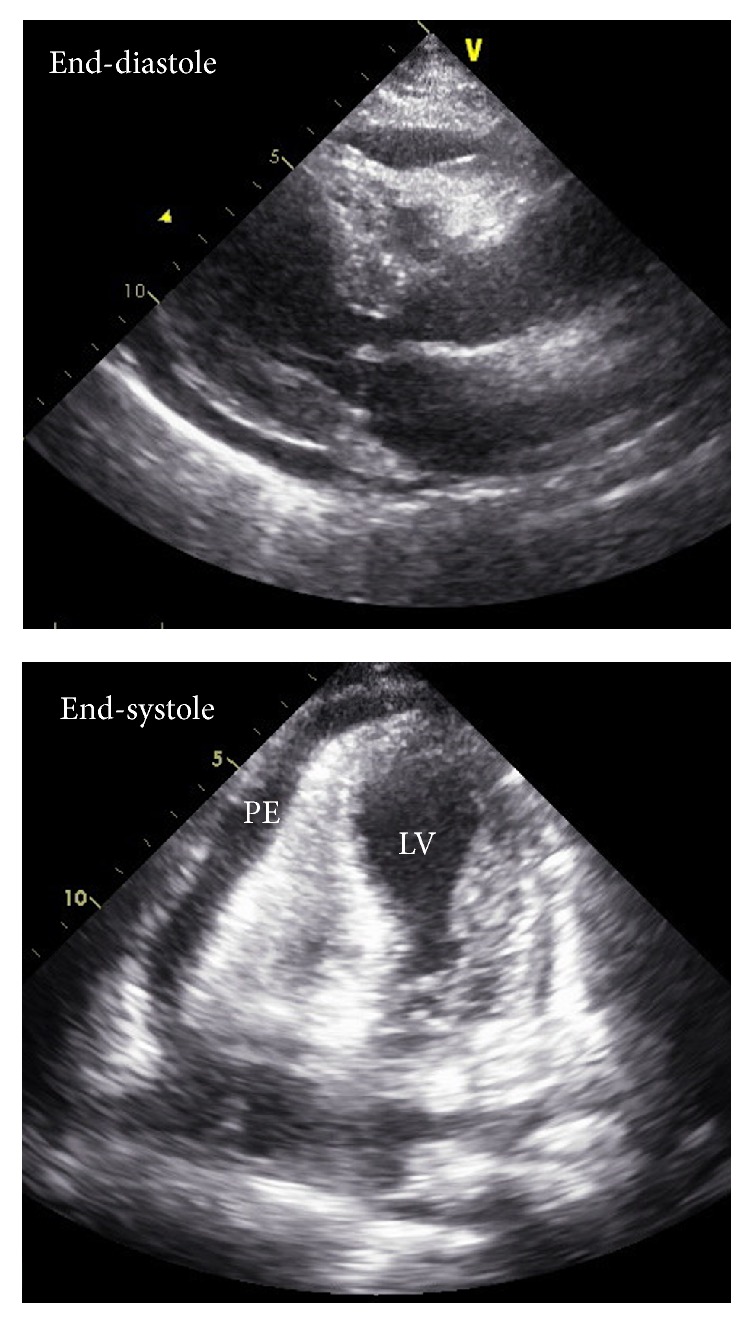
Transthoracic echocardiography showed small amounts of pericardial effusion, apical ballooning, and right ventricular collapse at end-diastole.

Three days after her readmission, the patient's condition had abruptly deteriorated involving a shock state and respiratory failure. Her vital signs were as follows: a blood pressure of 84/69 mm Hg, a heart rate of 142 bpm, and a respiration rate of 37 bpm. There was no symptom or sign indicating dehydration. Inotropic infusion and mechanical ventilation were applied. TTE revealed a small amount of pericardial effusion with right ventricular collapse during late-diastole and dilated inferior vena cava without respiratory variation (Figure 4). LVOT obstruction was aggravated to a peak velocity over 5 m/s. Immediate therapeutic pericardiocentesis and fluid infusion were performed to treat for cardiac tamponade, after which the patient's blood pressure promptly recovered up to 120/60 mm Hg. She achieved hemodynamic stability without inotropic support by the next day. Pericardial effusion was exudate (protein 5.7 g/dl, LDH 2587 U/L, neutrophil 52%, and lymphocyte 31%), but there was no evidence of malignancy or infection. Her condition gradually improved and follow-up TTE after 2 weeks revealed spontaneously recovered LV systolic function without wall motion abnormality.

## 3. Discussion

TCM is characterized by transient LV dysfunction, ECG changes, and elevation of cardiac markers, similar to presentation of ACS, and it occurs following emotional or physical stress. In addition to physical stress from surgery or anesthesia, physical stress from cardiology procedures may trigger TCM [5, 7]. In the present case, TCM was triggered by physical stress induced by CAG and ergonovine provocation test. The main pathophysiology of TCM is catecholamine-induced microvascular vasoconstriction and direct myocardial toxicity [8, 9]. The epicardial vasospasm was proposed as one of the mechanisms of TCM. However, this hypothesis has not been confirmed. In the present case, vasospasm by ergonovine provocation test was confirmed. However, there were no vasospasm on the second CAG and no response to nitroglycerine. Therefore, we believe that TCM, not myocardial infarction, was the cause of readmission.

The clinical course and prognosis of TCM are generally favorable, but several complications can occur including heart failure, arrhythmia, cardiogenic shock, LVOT obstruction, mitral regurgitation, ventricular thrombus, cardiac rupture, and death [10]. While many studies report good long-term prognosis, the acute phase of TCM can be life-threatening [1]. In the present case, the patient experienced a fatal complication, cardiac tamponade with LVOT obstruction. Cardiac tamponade and LVOT obstruction were interrelated in this case. A disproportionate increase in the pericardial pressure even smaller increments in pericardial fluid can cause a cardiac tamponade. Moreover, depending on anatomical variations and the susceptibility of the patient, acute LVOT obstruction can occur, which can further exacerbate the development of acute failure syndrome [11]. In the present case, extensive apical ballooning and a hyperkinetic basal wall may have contributed to aggravation of LVOT obstruction although she was of euvolemic status. And low cardiac output caused by LVOT obstruction can then further exacerbate cardiac tamponade.

In conclusion, this is, to the best of our knowledge, the first report of TCM induced by CAG with ergonovine test and complicated with cardiac tamponade. It is important, therefore, to note that CAG with provocation test can trigger TCM and that fatal complications can result. Furthermore, in conditions of low cardiac output, small amount of pericardial effusion can be presented as cardiac tamponade. These factors should be considered during acute phase management of TCM.

## References

[1] Ghadri J. R., Ruschitzka F., Luscher T. F. (2014). Takotsubo cardiomyopathy: still much more to learn. *Heart*.

[2] Scantlebury D. C., Prasad A., Prasad A. (2014). Diagnosis of takotsubo cardiomyopathy. *Circulation Journal*.

[3] Akashi Y. J., Nef H. M., Möllmann H., Ueyama T. (2010). Stress cardiomyopathy. *Annual Review of Medicine*.

[4] Tsuchihashi K., Ueshima K., Uchida T. (2001). Transient left ventricular apical ballooning without coronary artery stenosis: a novel heart syndrome mimicking acute myocardial infarction. *Journal of the American College of Cardiology*.

[5] Dias A., Franco E., Usatii V. (2013). Stress-induced cardiomyopathy shortly after pacemaker placement. *Journal of Invasive Cardiology*.

[6] ter Bals E., Odekerken D. A. M., Somsen G. A. (2014). Takotsubo cardiomyopathy complicated by cardiac tamponade. *Netherlands Heart Journal*.

[7] Lee J.-W., Kim J.-Y., Youn Y.-J. (2010). Clinical characteristics and prognostic factors of stress-induced cardiomyopathy. *Korean Circulation Journal*.

[8] Lyon A. R., Rees P. S. C., Prasad S., Poole-Wilson P. A., Harding S. E. (2008). Stress (Takotsubo) cardiomyopathy—a novel pathophysiological hypothesis to explain catecholamine-induced acute myocardial stunning. *Nature Clinical Practice Cardiovascular Medicine*.

[9] Akashi Y. J., Nef H. M., Lyon A. R. (2015). Epidemiology and pathophysiology of Takotsubo syndrome. *Nature Reviews Cardiology*.

[10] Jaguszewski M., Fijalkowski M., Nowak R. (2012). Ventricular rupture in Takotsubo cardiomyopathy. *European Heart Journal*.

[11] Redfors B., Ali A., Shao Y., Lundgren J., Gan L.-M., Omerovic E. (2014). Different catecholamines induce different patterns of takotsubo-like cardiac dysfunction in an apparently afterload dependent manner. *International Journal of Cardiology*.

